# Optimization of Embedded Sensor Packaging Used in Rollpave Pavement Based on Test and Simulation

**DOI:** 10.3390/ma15062283

**Published:** 2022-03-19

**Authors:** Zhoujing Ye, Yanxia Cai, Chang Liu, Kaiji Lu, Dylan G. Ildefonzo, Linbing Wang

**Affiliations:** 1 National Center for Materials Service Safety, University of Science and Technology Beijing, Beijing 100083, China; yezhoujing@ustb.edu.cn (Z.Y.); g20199178@xs.ustb.edu.cn (C.L.); 2 Beijing Zhonglu Gaoke Highway Technology Co., Ltd., Beijing 100088, China; 13311530926@163.com (Y.C.); kj.lu@rioh.cn (K.L.); 3 Research and Development Center of Transport Industry of New Materials, Technologies Application for Highway Construction and Maintenance, Beijing 100088, China; 4 Research Institute of Highway Ministry of Transport, Beijing 100088, China; 5 Center for Smart and Green Civil Systems, Virginia Tech, Blacksburg, VA 24060, USA; ildefonzo@vt.edu; 6 Joint USTB-Virginia Tech Lab on Multifunctional Materials, University of Science and Technology Beijing, Beijing 100083, China

**Keywords:** embedded sensor, Rollpave pavement, three-point bending test, dynamic response, finite element analysis, packaging optimization

## Abstract

Rollpave pavement, as a rollable prefabricated asphalt pavement technology, can effectively reduce the overall road closure time required for pavement construction and maintenance. Sensors can be integrated into Rollpave pavement, thereby avoiding sensor damage that may otherwise result from high temperatures and compactive forces during the rolling process, as well as pavement structural damage resulting from cutting and drilling. However, the embedment of sensors into Rollpave pavement still presents certain challenges, namely poor interfacial synergy between the embedded sensor and the asphalt mixture. To solve this problem, three-point bending tests and dynamic response FEM simulations were used to optimize the embedded sensor’s packaging. The influence of sensor embedment on Rollpave pavement under different working conditions was analyzed. Results of these analyses show that low temperature and the epoxy resin negatively affect the bending performance of specimens, and that packaging with cylindrical shape, flat design, and consisting of a material with modulus similar to that of the asphalt mixture should be preferred. This study is conducive to improve the intellectual level and service life of road infrastructure.

## 1. Introduction

With the increase of urban traffic volume, road construction and maintenance activities are more likely to cause serious traffic congestion and increased delays for the traveling public. In an effort to shorten road construction and maintenance time, Rollpave pavement, a new construction technology, was first proposed in the Netherlands [1]. Rollpave is a rollable pavement technology that is prefabricated in a manufacturing facility. During manufacturing, the pavement’s structural layers are fabricated, with specific functions, and then rolled up onto special reels for transport. Upon arrival at the construction site, the prefabricated pavement is unrolled like a carpet, allowing for rapid paving of the pavement layer [2,3]. Use of Rollpave technology can significantly improve the construction speed of asphalt pavement. Rollpave pavement has shown excellent potential as a pavement maintenance and repair technology [4,5].

The current research on Rollpave pavement mainly focuses on curling construction technology, new flexible pavement materials, and pavement structure functional integration. By studying the addition of new materials such as polyurethane, Wang et al. created Rollpave pavement with improved noise-reducing and anti-skid properties and improved the overall noise-reduction performance of this paving technology [6,7]. Steinauer explored the addition of energy harvesting and storage technologies for thermal, light, and piezoelectric energy in Rollpave pavement [8]. Guo et al. used high-elasticity asphalt to improve Rollpave pavement’s anti-fatigue and -permanent-deformation performance and developed supporting rolling equipment [9]. Dong et al. proposed a new type of modified asphalt for Rollpave mixtures [10]. Feng et al. proposed a “prefabricated asphalt pavement” for use in areas that cannot support construction requiring large-scale machinery [11]. Dai conducted research on the structure, performance, construction technology, and noise reduction capability of Rollpave pavement [12].

Rollpave pavement shows great technical advantages, especially in the ease of sensor embedment, a unique advantage for enabling the development of intelligent roadways. Prefabrication allows for more accurate placement of sensors within the pavement material, reduces the risk of sensor damage caused by high temperatures and forces from rolling and compaction, and removes the need for cutting and drilling of the pavement for sensor installation. When embedded with sensors, Rollpave pavement will have sensing functions for monitoring traffic information and evaluating the performance of the pavement structure. However, sensor embedment can adversely affect the performance of asphalt pavement structures, due to poor interfacial coupling between the sensor and asphalt mixture. Additionally, the evaluation metrics and experimental schemes for sensor coupling with the asphalt mixture interface are not presently well-defined [13,14]. In light of this fact, the evaluation of the influence of sensor embedment on Rollpave pavement and optimization of the sensor embedment scheme constitute topics of great research significance.

In this paper, a three-point bending test is conducted on asphalt beam specimens with embedded sensors, and the effect of sensor embedment on the bending performance of Rollpave asphalt mixture beam specimens is evaluated. A computational simulation of the dynamic response of Rollpave pavement with embedded sensors under moving vehicle loads is created. The results of this simulation are used to analyze the time-dependent characteristics of the maximum sensor–mixture interfacial stress under various operating conditions. Taken together, this paper illustrates the influence of sensor embedment on Rollpave pavement, with the aim of providing a reference for sensor packaging optimization. Improvement of sensor integration in Rollpave pavements will pave the way for future intelligent roadways with prolonged service lives.

## 2. Three-Point Bending Test by the Embedded Sensor

As Rollpave pavement requires prefabrication off-site, storage and transportation must be considered during manufacturing. Presently, the preferred method to accommodate storage and transportation is to form a thin, prefabricated asphalt layer and roll it into a cylindrical shape. However, in this form, the pavement structure will experience tensile and compressive stresses. To ensure structural performance during storage, it is necessary to evaluate the bending performance of the Rollpave pavement, as well as the impact of sensor embedment. The three-point bending test is suitable for determining the mechanical properties of the bending failure of asphalt mixtures at specified temperatures and loading rates. Therefore, the three-point bending test is performed on the asphalt mixture beam specimens, in order to evaluate the effect of the embedded sensor on the bending performance of Rollpave pavement.

### 2.1. Test Conditions

Several tests were conducted to analyze the effects of different factors on the bending performance of asphalt mixture beam specimens. Testing conditions are summarized in Table 1. The influence factors (independent variables) analyzed include sensor embedment, sensor size, sensor shape, sensor packaging materials, ambient temperature, and bonding materials.

### 2.2. Specimen Preparation

#### 2.2.1. Preparation of Asphalt Mixture

In this experiment, the asphalt mixture used to prepare the Rollpave pavement specimens has an oil-stone ratio of 7%, basalt as coarse aggregate, machine-made sand as fine aggregate, and bitumen penetration grade 70 as asphalt binder. The specific gradation of the mixture used is shown in Table 2.

**Figure 1:**
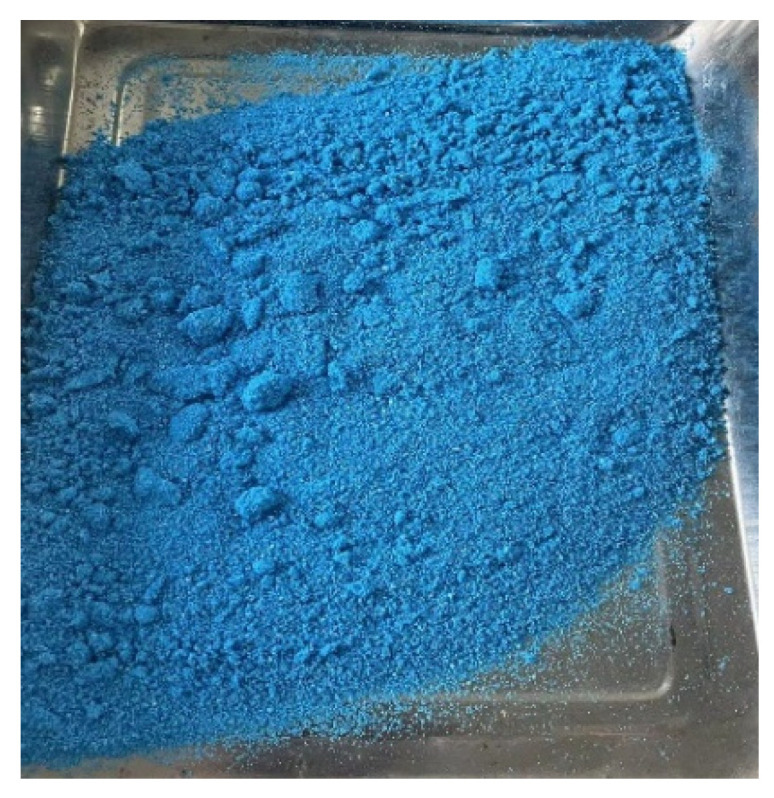
High-viscosity additive.

For the preparation of the asphalt mixture especially used for Rollpave pavement, high-viscosity additive (HVA) with 12% base asphalt, as shown in Figure 1, is added to mixed and stirred well. HVA can improve the asphalt viscosity and increase inter-aggregate bond strength, resulting in improved resistance to rutting and fatigue, low temperature crack resistance, and overall asphalt mixture stability.

#### 2.2.2. Sensor Packaging

**Figure 2:**
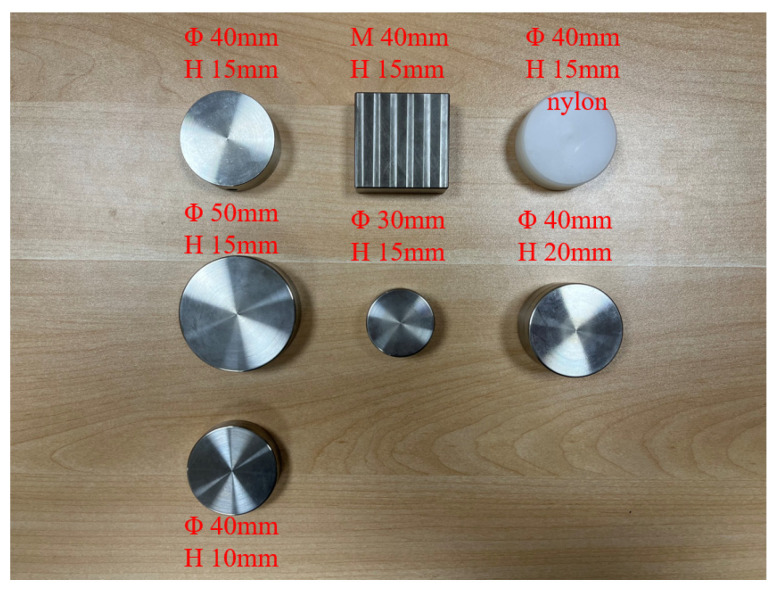
Different sensor packages.

Different sensor packages were designed and fabricated for the experiments. The different sensor packages used are shown in Figure 2. Packaging materials are composed of either 304 stainless steel or cast nylon. Package shapes are either cylindrical or cuboid, and sensor sizes cover different diameters and thicknesses. The dimensions of the packaging are mainly determined by both the size of the sensor chip and limitations of packaging processing technology. The packaging, as designed, can be used to protect built-in sensor chips, including MEMS accelerometers, temperature sensors, humidity sensors, vibration sensors, pressure sensors, and displacement sensors [15,16].

#### 2.2.3. Fabrication of Beam Specimen

**Figure 3:**
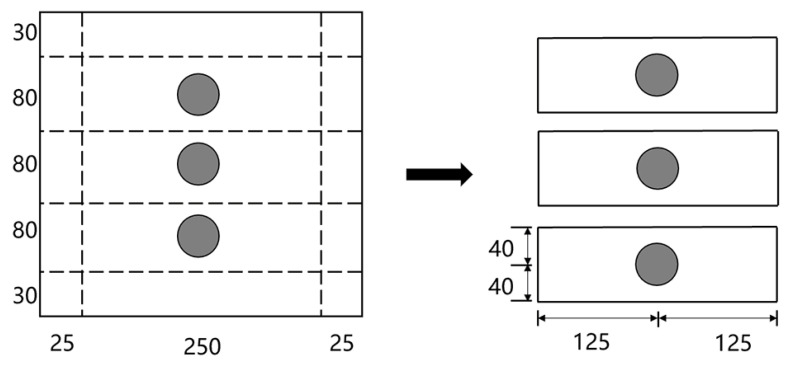
Dimension of beam specimen (unit: mm).

**Figure 4:**
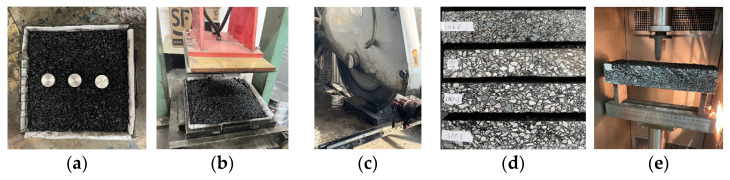
Beam specimen preparation. (**a**) Sensor positioning; (**b**) rutting plate specimens; (**c**) cutting of specimens; (**d**) beam specimens marking; (**e**) a three-point bending test.

During asphalt mixing, rutting plate specimens with embedded sensors are fabricated. The specific process for the fabrication of rutting plates is as follows: (1) the mold is preheated and the bottom and sides of the mold are brushed with oil; (2) paper is installed in the mold; (3) the asphalt mixture is evenly placed into the mold with a small shovel, and it is ensured that the center is slightly higher than the sides; (4) after placing the sensor in the designated position, the remaining asphalt mixture is added, and then a small, preheated hammer is used to tamp and level the mixture from the edges to the center. The rutting plate specimens are then cut into beam specimens for the three-point bending tests, as shown in Figure 3 and Figure 4.

In consideration of the boundary effect, the sensor is embedded in the center of the rutting plate specimen (sensors are embedded 1.25 cm away from the specimen surface) to maintain a certain distance from the specimen boundary [17]. According to the *Standard Test Methods of Bitumen and Bituminous Mixtures for Highway Engineering (JTG E20-2011)*, the rutting plate specimens are cut into beam specimens, with dimensions of 250 × 80 × 40 mm (length × width × thickness). The beam specimens are then placed in a temperature-controlled chamber, where they are kept at the set temperature for no less than four hours, until the internal temperature reaches ±0.5 °C of the test temperature. Once the test temperature is reached, the test specimen is taken out and placed on the supports of the three-point bending test machine. The distance between the fulcrum points was determined to be 200 ± 0.5 mm, so that the upper pressure head and the lower pressure head were kept parallel, and the two sides were equidistant. The hydraulic press then applies a concentrated load at the span center, at a loading rate of 50 mm/min, until specimen fracture occurs, at which point the specimen is considered to have failed. Three beam specimens were made for each working condition. Based on the average load and displacement of the three specimens, the load–midspan deflection curve for each case is obtained.

### 2.3. Evaluation Index of Bending Performance

**Figure 5:**
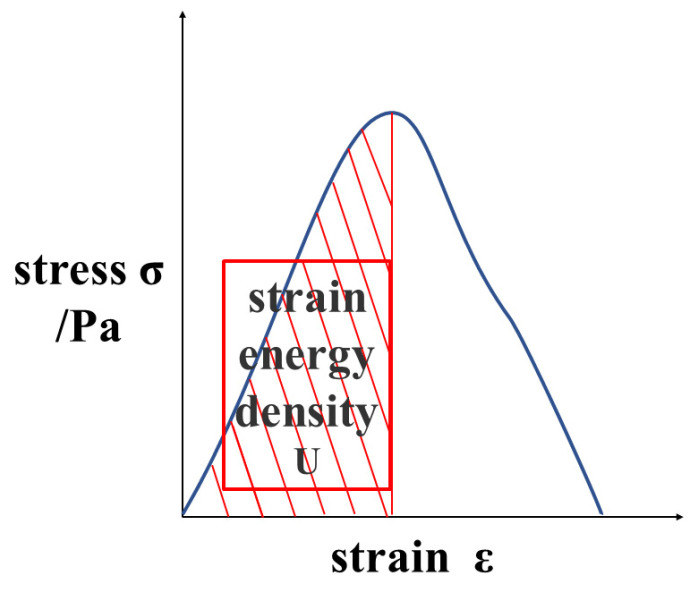
Bending strain energy.

As an index to measure the critical failure of specimens, flexural tensile strength and strain have opposite trends, making it challenging to quantitatively evaluate the bending performance by using both. Therefore, the bending strain energy is chosen as the evaluation metric [18]. The bending strain energy refers to the area enclosed by the stress–strain curve, and the *x*-axis before the stress reaches the peak value; this concept is illustrated in Figure 5. The unit of strain energy is KJ/m^3^. The bending strain energy characterizes the energy absorbed by the specimen before failure. The greater the bending strain energy, the greater the energy required to fail the specimen.

According to the load–displacement curve and Equations (1) and (2), the stress–strain curve is calculated. In addition, flexural tensile strength $R_{B}$, beam bottom maximum flexural tensile strain $\varepsilon_{B}$ at bending failure, and bending stiffness modulus $S_{B}$ can be calculated according to Equations (3)–(5):

(1) $$\sigma_{B}=\frac{3FL}{2bh^{2}}$$

(2) $$\varepsilon_{B}=\frac{6hd}{L^{2}}$$

(3) $$R_{B}=\frac{3P_{B}L}{2bh^{2}}$$

(4) $$S_{B}=\frac{R_{B}}{\varepsilon_{B}}$$

(5) U=∫ σdε

where F is the load; $P_{B}$ is the maximum load when the specimen fails; *h* is the height of the specimen; *B* is the width of the specimen; *d* is the midspan deflection when the specimen is loaded; *L* is the span length of the specimen; *U* is the bending strain energy; and all other variables are as designated above.

In consideration of the curling process of Rollpave pavement, the beam bottom maximum flexural tensile strain is selected as the evaluation index. The greater the beam bottom maximum flexural tensile strain of specimens before loading failure, the easier it is for the Rollpave pavement crimping process to be achieved. The bending stiffness modulus (Equation (4)) represents the ability of the beam specimens to resist bending deformation within elastic limit. The higher the value of bending stiffness modulus, the less effective the beam specimens are at resisting bending deformation within the elastic limit.

### 2.4. Influence Analysis of Bending Properties

**Figure 6:**
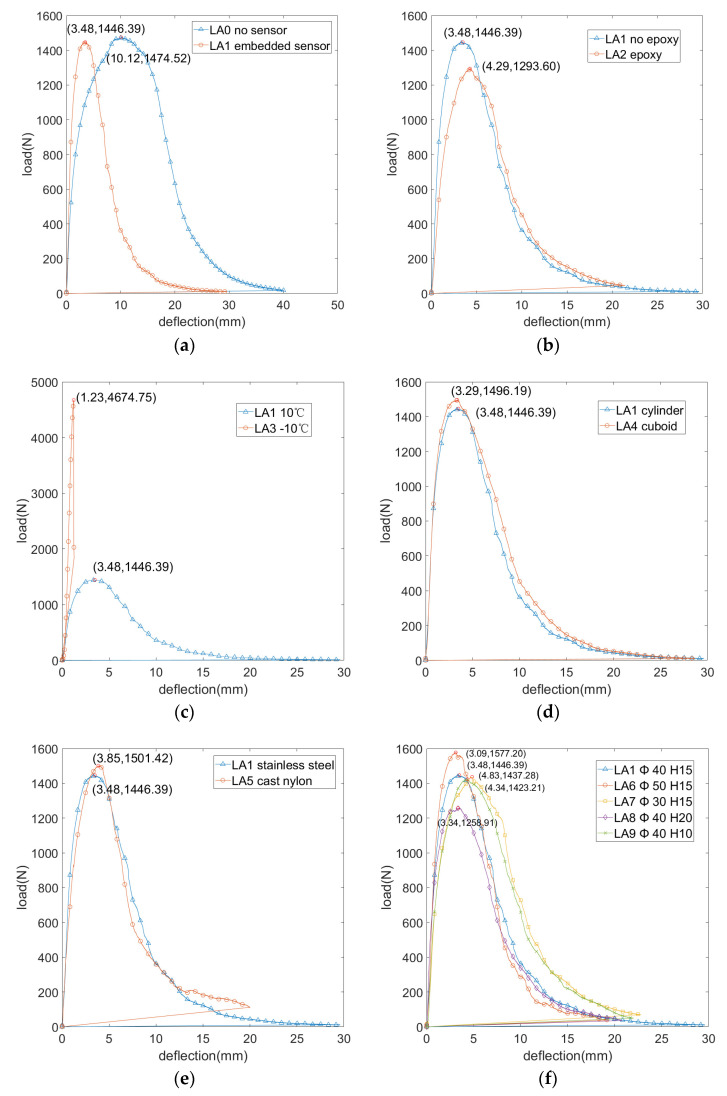
The load–midspan deflection curve under various test conditions. (**a**) Influence of embedded sensor; (**b**) influence of epoxy coating; (**c**) influence of temperature; (**d**) influence of sensor packaging shape; (**e**) influence of packaging material; (**f**) influence of sensor packaging size.

The load–midspan deflection curves of the specimens under various test conditions are obtained by the recorder attached to the three-point bending test frame and shown in Figure 6.

**Figure 7:**
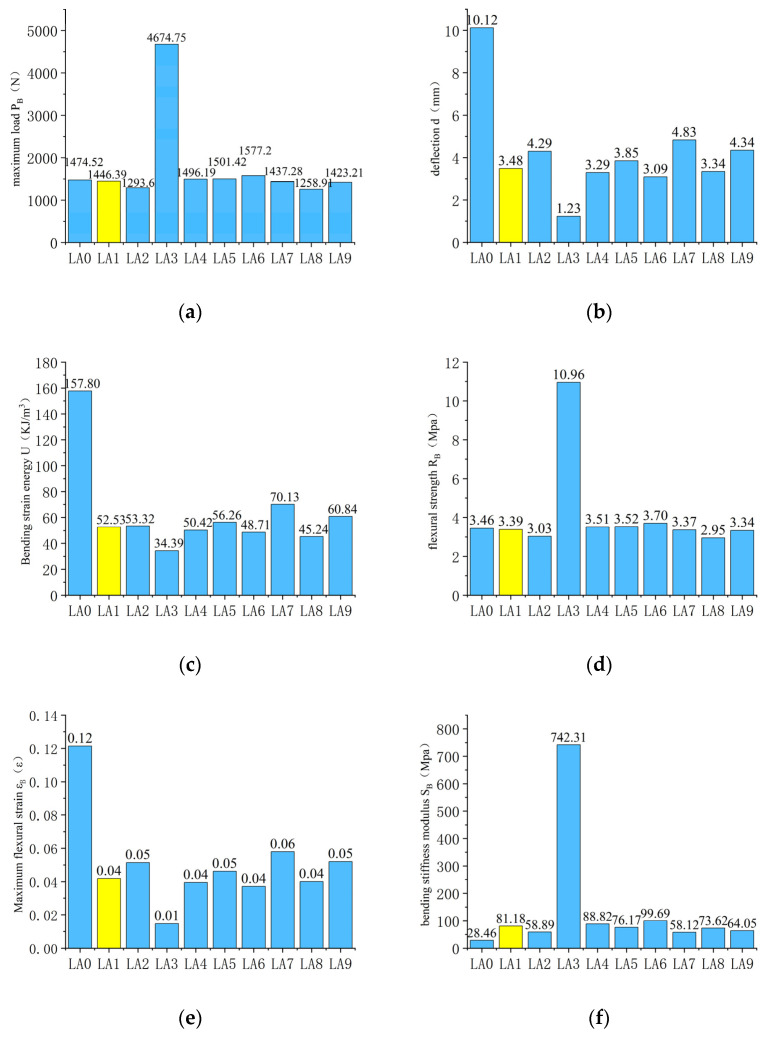
Evaluation index values for bending performance. (**a**) Maximum load *P_B_* at specimen failure; (**b**) mid-span deflection *d;* (**c**) bending strain energy *U*; (**d**) flexural tensile strength *R_B_*; (**e**) beam bottom maximum flexural tensile strain $\varepsilon_{B}$. (**f**) bending stiffness modulus $S_{B}$.

The values of the evaluation indices were calculated according to Equations (1) through (5), with testing condition LA1 set as the control group marked with yellow columns. The results are shown in Figure 7.

#### 2.4.1. Influence of Embedded Sensor

The influence of the embedded sensors on the bending performance of the beam specimens is analyzed by comparing test conditions LA0 and LA1. According to Figure 6a, it is shown that, with the increase of midspan deflection, specimens containing an embedded sensor experience a loading rate increase that is significantly higher than that of the specimen without an embedded sensor. The specimen containing an embedded sensor reached failure load in a shorter amount of time than did the specimen without an embedded sensor. According to Figure 6a, the maximum loads of the two tests are only slightly different, with maximum load rates of 1474.52 and 1446.39 N for testing conditions LA0 and LA1, respectively. Despite the small difference in maximum loading, the midspan deflections experience differed greatly between the two specimens, with deflection of 10.12 and 3.48 mm for testing conditions LA0 and LA1, respectively. Sensor embedment is, thus, observed to decrease midspan deflection at beam specimen failure load by 65.58%. By comparing the evaluation index values of test conditions LA0 and LA1 from Figure 7c, the bending strain energy of beam specimens decreased by 66.71%, from 157.8 to 52.53 KJ/m^3^ after embedding the sensor. According to Figure 7e, it was observed that the embedment of sensors makes beam specimens more prone to damage. There is little difference between flexural tensile strength for testing conditions LA0 and LA1. The beam bottom maximum flexural tensile strains for testing conditions LA0 and LA1 are 0.12 and 0.04 ε, respectively. According to Figure 7f, the bending stiffness modulus of condition LA1 is 81.18 MPa, greater than 28.46 MPa of condition LA0. It is, therefore, observed that the ability of the specimen with an embedded sensor to resist bending deformation is diminished, compared to the specimen without an embedded sensor.

**Figure 8:**
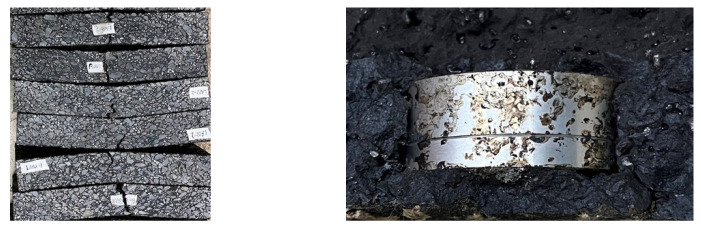
Specimen failure after the three-point bending test.

The sensor is embedded in the midspan of the beam specimen, and it is known that the stiffness of the sensors is greater than that of the asphalt mixture, making it more difficult to produce local deformation of the sensor. Therefore, under the same midspan deflection, the deformation of specimens with a sensor embedded is greater than that of specimens without a sensor embedded. There exists a stress concentration at the interface between the sensor and asphalt mixture, which will lead to the compression peeling of the sensor surrounding the asphalt mixture, as shown in Figure 8.

#### 2.4.2. Influence of Epoxy Coating

The influence of epoxy resin bonding on the bending performance of beam specimens is analyzed by comparing test conditions LA1 and LA2. According to Figure 6b, the failure load of the beam specimens with epoxy resin (test condition LA1) is 1446.39 N, while the failure load of beam specimens (testing condition LA2) is 1293.60 N; such a small difference in failure load is deemed insignificant. The load increase rates between conditions LA1 and LA2 are similar, and the corresponding deflections are 3.48 and 4.29 mm for LA1 and LA2, respectively. According to Figure 7c, conditions LA1 and LA2 have similar bending strain energy values, 52.53 and 52.32 KJ/m^3^, respectively. According to Figure 7d,e, the values of flexural tensile strength and beam bottom maximum flexural tensile strain are similar for both test conditions. According to Figure 7f, the bending stiffness modulus of LA1 is 81.18 MPa, which is greater than the 58.89 MPa bending stiffness modulus of LA2. It is, thus, observed that epoxy resin as a binder can indeed enhance the bending performance of specimens, but the effects are not significant. It is additionally understood that the adhesion between the asphalt mixture and sensor is good at high temperatures.

#### 2.4.3. Influence of Temperature

By comparing testing conditions LA1 and LA3, the effect of temperature on the beam specimens’ bending performance is analyzed. Figure 6c shows that temperature has a significant impact on the bending performance of beam specimens. When the loading temperature is −10 and 10 °C, the beam specimens failure loads are 1446.39 N and 4674.75 N, respectively. The corresponding midspan deflections are 3.48 and 1.23 mm for conditions LA1 and LA3, respectively. Figure 7 shows that the bending strain energies of LA1 and LA3 are 52.53 and 34.39 KJ/m^3^, respectively. Moreover, there are significant differences in flexural tensile strength, beam bottom maximum flexural tensile strain, and bending stiffness modulus.

At low temperatures, asphalt mixtures can become hard and brittle. As the bending stiffness modulus increases, the resistance to bending deformation within the elastic limit decreases significantly. Therefore, the load at failure is larger, and the corresponding deflection is smaller. For bending strain energy, the energy required for specimens to fail at low temperature is lower, meaning that the specimens are more likely to fail at a low temperature than they are at normal temperature. Therefore, low-temperature fabrication of Rollpave pavement with embedded sensors should be avoided.

#### 2.4.4. Influence of Sensor Packaging Shape

The influence of sensor shape (cylinder or cuboid) on the bending performance of beam specimens is analyzed by comparing testing conditions LA1 and LA4. According to Figure 6d, the failure load and deflection of beam specimens embedded with cylindrical and cuboid sensors are similar, and the corresponding midspan deflections are 3.48 and 3.29 mm for conditions LA1 and LA4, respectively. According to Figure 7c, cylindrical sensor packaging topology can increase the bending strain energy required for specimens to break to a small extent, but no significant difference is observed between the two specimens. No significant differences in the values of flexural tensile strength and beam bottom maximum flexural tensile are observed between testing conditions LA1 and LA4. According to Figure 7f, the bending stiffness modulus for conditions LA1 and LA4 are 81.18 and 88.82 MPa, respectively. Sensor packaging shape is observed to have little influence on the bending performance of beam specimens. To a small extent, the cylindrical topology of the sensor packaging can slightly improve the bending performance of specimens, but this does not constitute a significant improvement.

#### 2.4.5. Influence of Packaging Material

The influence of sensor materials (stainless steel or cast nylon) on beam specimen bending performance is analyzed by comparing testing conditions LA1 and LA5. Figure 6e shows that the failure loads of the beam specimens with embedded sensors in stainless steel packaging (condition LA1) is 1446.4 N, while the failure load of beam specimens embedded with sensors in cast nylon packaging is 1501.4 N. Midspan deflections are 3.48 and 3.85 mm for conditions LA1 and LA5, respectively. According to Figure 7, the bending strain energy of the specimen containing an embedded sensor packaged in cast nylon is 56.26 KJ/m^3^, while the bending strain energy of the specimen containing an embedded sensor packaged in stainless steel is 52.53 KJ/m^3^. Flexural tensile strength, beam bottom maximum flexural tensile strain, and bending stiffness modulus are all similar between the two specimens tested. The bending stiffness modulus of condition LA1 is 81.18 MPa, which is greater than 76.17 MPa, which is observed for condition LA5. The sensor material is, thus, thought to have little influence on the bending performance of beam specimens, though the use of cast nylon sensor packaging can improve the bending performance of specimens to some extent. It is hypothesized that improvements seen in specimens with cast nylon sensor packaging are owed to the fact that the elastic modulus of nylon is closer to that of the road surface, as well as the fact that the nylon surface is rougher than the surface of the stainless steel, which can create better adhesion at the sensor–asphalt interface.

#### 2.4.6. Influence of Sensor Packaging Size

By comparing the LA1, LA6, LA7, LA8, and LA9 test conditions, the impact of the sensor size is analyzed in two ways. First, the impact of the sensor diameter is analyzed by selecting three beam specimens containing sensors with packaging of the same thickness but different diameters. In this case, beam specimens LA1, LA6, and LA7 are selected for comparison, as they all have sensors of 15 mm thickness. Figure 6f shows that the midspan deflection at failure for specimens containing embedded sensors with diameters of 30, 40, and 50 mm (corresponding to specimens LA7, LA1, and LA6) are 4.83, 3.48, and 3.09 mm, respectively. Next, the impact of sensor thickness is analyzed by selecting three beam specimens containing sensors with packaging of the same diameter but different thicknesses. In this case, beam specimens LA1, LA8, and LA9 are selected for comparison as they all have sensors of 40 mm diameters. The midspan deflection at failure for beam specimens with sensor thicknesses of 10, 15, and 20 mm (corresponding to specimens LA9, LA1, and LA8) are 4.34, 3.48, and 3.34 mm, respectively. According to Figure 7, based on the values of bending strain energy and bending stiffness moduli across testing conditions, it is observed that specimens containing sensor packaging of both smaller diameter and thickness have higher strain energy at failure. That is, smaller embedded sensor packaging results in stronger resistance to deformation in the beam specimen. It is, therefore, necessary to reduce the sensor package size as much as possible, in order to improve the bending performance of the pavement.

## 3. Dynamic Response Simulation of Rollpave Pavement

In the three-point bending test, the loading force is fundamentally different from that of a road surface under vehicle load [19]. The loading force of three-point bending test is concentrated force, while the loading force of vehicle is a uniform load. In addition, the specimen size of three-point bending test is also different from the real road surface, which may lead to differences in experimental results. To further explore the influence of embedded sensors on Rollpave pavement, a simulation of the dynamic response of Rollpave pavement under vehicle moving loading is carried out.

### 3.1. Model Establishment

#### 3.1.1. Pavement Structure and Materials

Referring to the pavement structure of the National Center for Materials Service Safety (NCMS) test road, The elastic modulus, Poisson’s ratio, and damping ratio were derived from *the Specifications for Design of Highway Asphalt Pavement (JTG D50-2017)* [20]. The pavement structure and material parameters are shown in Table 3, in which, the upper layer is Rollpave pavement.

#### 3.1.2. Model Size and Mesh Generation

**Figure 9:**
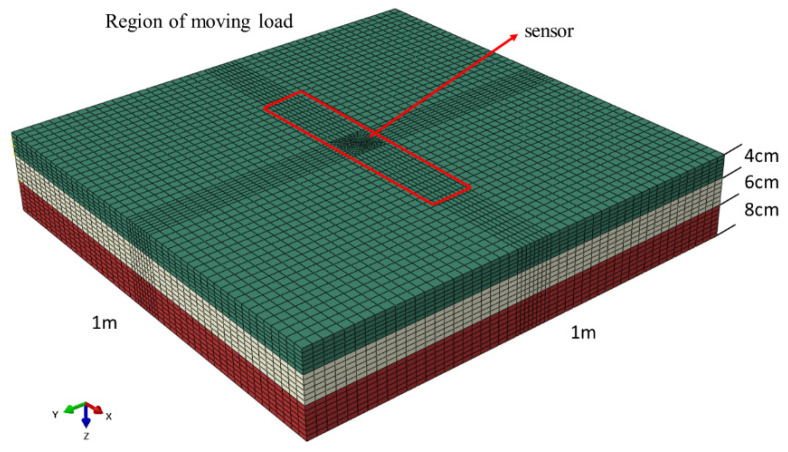
A 3D model of Rollpave pavement with an embedded sensor.

A 3D finite element model (FEM) of Rollpave pavement with embedded sensors is established using Abaqus software, as illustrated in Figure 9. The road model is of dimensions 1.0 × 1.0 × 0.18 m (length × width × depth). The driving direction is taken to be the same as that of the positive *x*-axis, and the vertical direction is taken to be the same as that of the positive *z*-axis. The cylindrical sensor of dimensions 40 (diameter) and 15 mm (height) is centered on the Rollpave pavement. The top of the sensor is 1.25 cm from the road surface, the bottom is 1.25 cm from the bottom of the Rollpave pavement, and the sides are 0.48 m from the road boundary, reducing the influence of the model boundary on the sensor stress analysis.

The road model was idealized using linear hexahedral elements of type C3D8R. The length and width of elements were set as 2 × 2 cm. The meshes of the loading area were refined, and the model was meshed into 54,986 elements.

#### 3.1.3. Constrain Condition and Moving Load

The three direction movements and rotations were restrained at the bottom of the model, and the normal directions were restrained, corresponding to the four sides of the model. Uniaxial vehicle loads are idealized as moving uniform rectangular loads and imparted on the model, using the Abaqus subroutine DLOAD module [21]. The loading area was set to be in the middle of the model. The length of the moving load area was set to 0.5 m, and the width to 0.216 m. Three moving load working conditions with magnitudes of 0.7, 1.4, and 2.1 MPa were considered. Further, working conditions with loading speeds of 10 and 20 m/s was considered. The most unfavorable working condition was selected as the loading condition of the subsequent simulation.

### 3.2. Error Analysis of Mesh Generation

**Figure 10:**
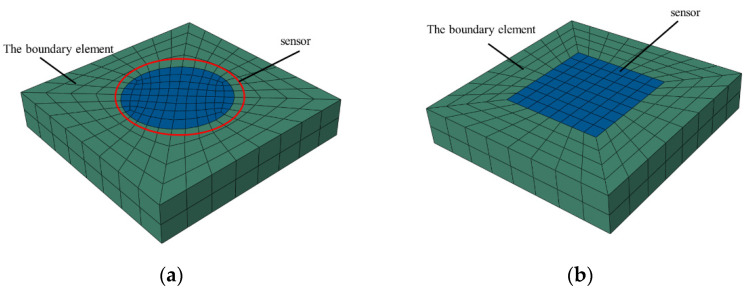
Mesh generation of sensors with different shapes. (**a**) Embedded cylinder sensor; (**b**) embedded cuboid sensor.

Due to the difference in the mesh generation between cylindrical and cuboid sensor shapes, the element shape and position at the interface are also different, which may result in errors in subsequent stress analyses. An error analysis of the mesh generation is performed during subsequent stress analyses to correct for errors arising from differences in mesh generation. Figure 10 shows the mesh generation results for the cuboid and cylindrical sensors. The cylindrical sensor is 40 mm in diameter and 15 mm in height, and the cuboid sensor is 40 mm in length and width and 15 mm in height.

**Figure 11:**
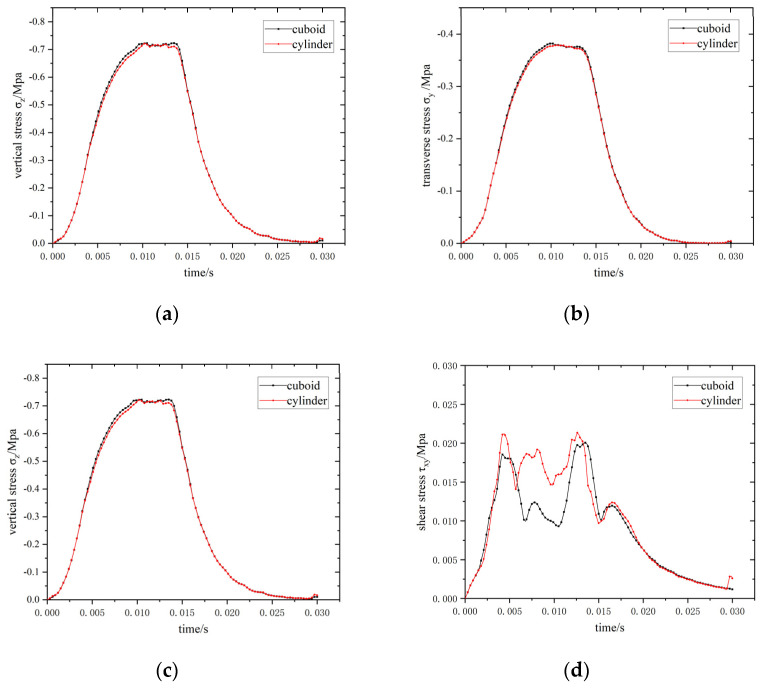

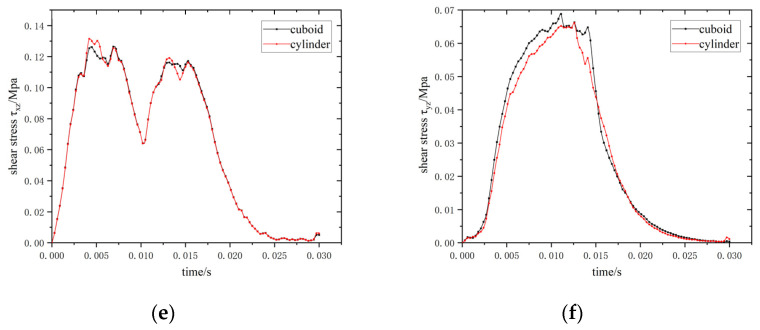
Maximum stress–time curves of elements at the interface. (**a**) Longitudinal stress (σ_x_); (**b**) transverse stress (σ_y_); (**c**) vertical stress (σ_z_); (**d**) shear stress (τ_xy_); (**e**) shear stress (τ_xz_); (**f**) shear stress (τ_yz_).

After the meshes are generated for the different sensor packaging shapes, other variables are controlled. By setting the sensor material properties (the pavement materials), boundary conditions (merge connection with pavement elements), and moving loads (0.7 MPa, 20 m/s) to the same working conditions, the maximum stress–time curves of the elements at the interface under different mesh generations are compared, as illustrated in Figure 11.

No significant difference is observed between the maximum stresses of elements at the material interface during moving load between the two mesh types. The existing two types of mesh generation can, thus, be used to analyze the stress. No large error, caused by differences in the meshes, are observed.

Under vehicle load, the maximum normal stress in the pavement structure is vertical stress, σ_z_, and the maximum shear stress is τ_xz_ (shear stress along the axis of the driving direction). Therefore, vertical stress, σ_z_, and shear stress, τ_xz_, are selected as the parameters for evaluating the stress concentration at the interface between the sensor and pavement material. Greater values of the selected parameters correspond to higher stress concentrations at this interfacial boundary.

### 3.3. Sensor Packaging Optimization

The mechanical properties of sensor packaging materials are different than those of the pavement materials. The maximum vertical stress, σ_z_, and shear stress, τ_xz_, in all elements at the interface are used to evaluate the synergistic performance between the embedded sensor and asphalt mixtures and are used to generate the maximum stress–time curves. Additionally, sensor packaging optimization is carried out for different driving conditions and sensor packaging shapes, materials, and sizes, as shown in Table 4.

#### 3.3.1. Comparison of Driving Conditions

**Figure 12:**
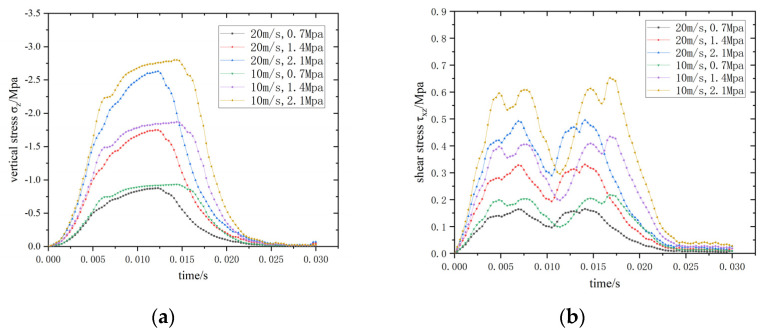
Maximum stress–time curve of elements at the interface under different driving conditions. (**a**) σ_z_-time; (**b**) τ_xz_-time.

The most unfavorable working conditions for the synergistic performance between the pavement structure and embedded sensor are analyzed under various driving conditions. Driving conditions include combinations of vehicle speed and load magnitudes and are composed of vehicle speeds of 10 and 20 m/s, with load magnitudes of 0.7, 1.4, and 2.1 MPa. Figure 12 shows the maximum stress–time curves of elements at the interface under different driving conditions.

The maximum vertical stress and maximum shear stress of the elements at the interface increase, both as the amplitude of the moving load increases and velocity of the moving load decreases. The combination of a speed of 10 m/s and moving load amplitude of 2.1 MPa represent the most unfavorable condition. Accordingly, this combination is adopted for the subsequent optimization analysis.

#### 3.3.2. Comparison of Sensor Shapes

**Figure 13:**
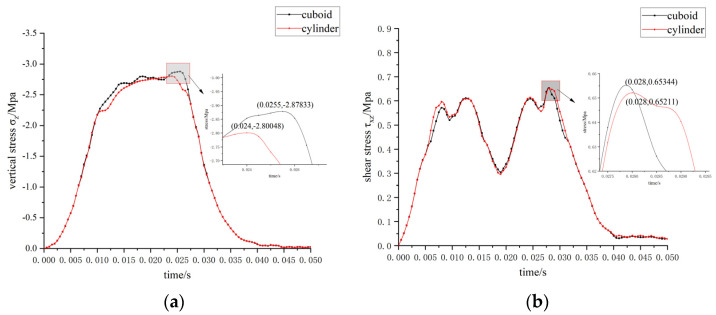
Maximum stress–time curve of elements at the interface under different sensor shapes. (**a**) σ_z_-time; (**b**) τ_xz_-time.

Analysis of the impact of sensor packaging shape on mechanical performance is completed by comparing the cuboid sensor packaging with the cylindrical sensor packaging. In both cases, stainless steel is selected as the packaging material. The driving condition is selected as previously described. Figure 13 shows the maximum stress–time curve of elements at the interface under different sensor shapes.

Since the sensor is small, relative to the size of the road model, a difference in sensor packaging shape has little effect on the results of the stress analysis. Compared to the cuboid sensor packaging, the cylindrical sensor packaging performs only slightly better at low speed and heavy load. The maximum vertical stress of the cylindrical sensor packaging is reduced by 2.7%, compared to that of the cuboid sensor packaging, and the maximum shear stress is reduced by 0.2%. Therefore, the cylindrical packaging is selected as the optimum sensor packaging shape.

#### 3.3.3. Comparison of Sensor Materials

**Figure 14:**
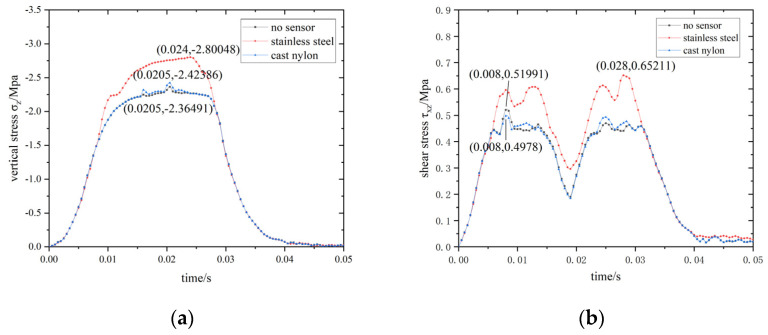
Maximum stress–time curve of elements at the interface under different sensor materials. (**a**) σ_z_-time; (**b**) τ_xz_-time.

An analysis of sensor packaging materials was completed. Packaging materials consider the sensor include stainless steel and cast nylon, the material parameters of which are shown in Table 5. The maximum stress–time curve of elements at the interface, under different sensor materials, is shown in Figure 14.

When cast nylon is used as the sensor packaging material, both vertical and shear stress are closest to the pavement without an embedded sensor. Maximum vertical stress under the cast nylon packaging is reduced by 13.45%, and the maximum shear stress is reduced by 23.66%, when compared to the stainless steel sensor packaging. Therefore, cast nylon is observed to be the more optimum of the two sensor packaging materials.

#### 3.3.4. Comparison of Sensor Sizes

**Figure 15:**
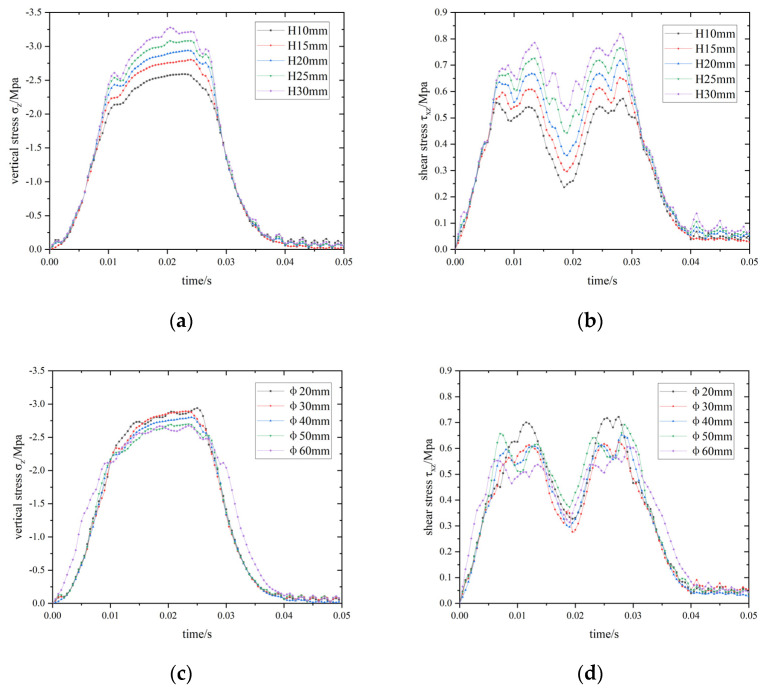
Maximum stress–time curve of elements at the interface under different sensor sizes. (**a**) σ_z_-time curve, sensor thicknesses; (**b**) τ_xz_-time curve, sensor thicknesses; (**c**) σ_z_-time curve, sensor diameters; (**d**) τ_xz_-time curve, sensor diameters.

An analysis on the effect of sensor packaging size on stress concentration in Rollpave pavement was completed. First, the effects of different sensor thicknesses are compared and analyzed by holding the sensor packaging diameter constant at 40 mm. Second, the effects of varying sensor packaging diameters are compared and analyzed by holding sensor thickness constant at 15 mm. The results of these analyses are shown in Figure 15.

With the sensor diameter held constant, both the maximum vertical stress and shear stress decrease with decreasing sensor thickness. It, thus, follows that the smaller the sensor thickness, the less stress concentration. However, the maximum vertical stress decreases as the sensor diameter increases, and there is no obvious correlation between the maximum shear stress and sensor diameter.

For the above simulation, it should be noted that the connection between the road and sensor elements at the interface is set to “Merge” in the ABAQUS program; thus, the nodal displacements at the interface are always consistent. However, in reality, debonding may occur at the interface between the sensor and asphalt mixture.

## 4. Conclusions

In this paper, the influence of embedded sensors in Rollpave pavement are analyzed via three-point bending test and dynamic response simulation, and the embedded sensor packaging used in Rollpave pavement are optimized. The conclusions are as follows:

(1) Bending strain energy, the amount of energy absorbed by the specimens before destruction, was found to be an effective parameter for the characterization of the bending performance of beam specimens containing an embedded sensor. Under the action of vehicle loading, stress concentrations will appear at the interface between the sensor and asphalt mixture. The maximum normal stress is vertical stress, σ_z_, and the maximum shear stress is in the x-z plane, τ_xz_; these parameters can be used to evaluate the stress concentration level at the sensor-pavement interface.

(2) The results of the three-point bending test show that the embedment of sensors significantly reduced beam specimens’ ability to resist bending deformation. At low temperatures, the failure load of the specimens increases, and the deflection decreases, making the specimens more prone to failure than they would be at normal temperature. Use of epoxy resin as an adhesive does not effectively enhance the bending performance of the specimens. Unexpectedly, it was found that, at high temperatures, the asphalt mixture bonded well to the sensor, even without the addition of an adhesive. Use of the cylindrical sensor packaging shape with cast nylon encapsulation, combined with the reduction of sensor size, was shown to increase the bending performance of the specimens.

(3) The simulation results show that the most unfavorable vehicle loading conditions for roadway structural health are the combination of low speed of travel and a heavy moving load. It was found that the selection of sensor packaging materials (e.g., nylon) with modulus, similar to the pavement materials, are better choices. Analysis of sensor size revealed that smaller and more circular sensor packaging exhibits better performance. Further, sensor thickness was observed to have a greater effect on bending performance than sensor diameter. For optimum performance, the use of a flat sensor packaging design and minimization of sensor packaging height are both recommended.

(4) In the future, drawing and shear tests should be carried out to evaluate the effect of the sensor surface texture on the bonding performance and better define the failure mechanism of the sensor–asphalt mixture interface. This study optimizes the embedded sensor packaging used in Rollpave pavement. The interfacial synergy between the embedded sensor and asphalt mixture can be improved, which is helpful to prolong the service life of intelligent roadways.

## Figures and Tables

**Table 1:** Designed test conditions.

No.	Packaging Material	Sensor Shape	Sensor Size	Bonding Material	Temperature	Influence Factor
LA0	Without embedding sensor	/	/	No adhesive	10 °C	Sensor embedment
LA1	Stainless steel	Cylinder	Φ 40 mm H 15 mm	No adhesive	10 °C	Control group
LA2	Stainless steel	Cylinder	Φ 40 mm H 15 mm	Epoxy	10 °C	Bonding materials
LA3	Stainless steel	Cylinder	Φ 40 mm H 15 mm	No adhesive	−10 °C	Temperature
LA4	Stainless steel	Cuboid	L 40 mm M 40 mm H 15 mm	No adhesive	10 °C	Shape
LA5	Cast nylon	Cylinder	Φ 40 mm H 15 mm	No adhesive	10 °C	Packaging material
LA6	Stainless steel	Cylinder	Φ 50 mm H 15 mm	No adhesive	10 °C	Sensor size (diameter)
LA7	Stainless steel	Cylinder	Φ 30 mm H 15 mm	No adhesive	10 °C	
LA8	Stainless steel	Cylinder	Φ 40 mm H 20 mm	No adhesive	10 °C	Sensor size (thickness)
LA9	Stainless steel	Cylinder	Φ 40 mm H 10 mm	No adhesive	10 °C	

**Table 2:** Gradation of composite aggregate.

	Cumulative Percent Passing Each Sieve (%)								
Aperture sizes (mm)	13.2	9.5	4.75	2.36	1.18	0.6	0.3	0.15	0.075
Gradation	100	93	47	30	20	17	15	12	10

**Table 3:** Pavement structure and material parameters.

No.	Structure	Thickness [cm]	Elastic Modulus [Mpa]	Poisson’s Ratio	Density [kg/m^3^]	Damping Ratio
1	Upper layer Rollpave pavement	4	1100	0.35	2500	0.05
2	Middle layer AC-20	6	1300	0.35	2400	0.05
3	Lower layer AC-25	8	1200	0.35	2400	0.05

**Table 4:** Multiple working conditions.

Set Conditions	Operating Conditions	Test Purpose
Shape: cylinder Size: φ40 * 15 mm Materials: stainless steel	Load magnitude: 0.7 Mpa, 1.4 Mpa, 2.1 Mpa Speed: 20 m/s, 10 m/s	Compare different loading conditions
Materials: stainless steel Load magnitude: 2.1 Mpa Speed: 10 m/s	Cuboid: 40 * 40 * 15 mm Cylinder: φ40 * 15 mm	Compare different sensor shapes
Shape: cylinder Size: φ40 * 15 mm Load magnitude: 2.1 Mpa Speed: 10 m/s	Stainless steel Cast nylon	Compare different sensor materials
Materials: stainless steel Shape: cylinder Load magnitude: 2.1 Mpa Speed: 10 m/s	Change in thickness 10/15/20/25/30 * φ40 mm Change in diameter Φ20/30/40/50/60 * 15 mm	Compare different sensor sizes

**Table 5:** Material parameters of different sensor packaging materials.

Material	Elastic Modulus (Mpa)	Poisson’s Ratio	Density (kg/m^3^)
Cast nylon	2500	0.35	930
Stainless steel	200,000	0.3	8000

## Data Availability

Data is contained within the article.

## References

[1] Houben L.J.M., van der Kooij J., Naus R.W.M., Bhairo P.D. APT Testing of modular pavement structure ‘Rollpave’ and comparison with conventional asphalt motorway structures. Proceedings of the 2nd International Conference on Accelerated Pavement Testing.

[2] Molenaar J.M.M., Montfort J. (2011). Super Stille Deklaag Rubber Rollpave (in Dutch).

[3] Dommelen A.E., Kooij J.V.D., Houben L.J.M., Molenaar A.A.A. LinTrack APT research supports accelerated implementation of innovative pavement concepts in the Netherlands. Proceedings of the 2nd International Conference on Accelerated Pavement Testing.

[4] Mao X., Zhou K. (2017). Rollpave Pavement Construction Technology and Its Development Trend. West. China Commun. Sci. Technol..

[5] Ingram L.S., Herbold K.D., Rasmussen R.O., Baker T.E., Brumfield J.W., Felag M.E., Ferragut T.R., Grogg M.G., Lineman L.R. (2004). Superior Materials, Advanced Test Methods and Specifications in Europe.

[6] Wang D., Schacht A., Chen X., Oeser M., Steinauer B. (2013). Feasibility study on the innovative construction method of a ‘prefabricated and rollable road’. BAUTECHNIK.

[7] Wang D., Schacht A., Chen X., Liu P., Oeser M., Steinauer B. (2016). Innovative Treatment to Winter Distresses Using a Prefabricated Rollable Pavement Based on a Textile-Reinforced Concrete. J. Perform. Constr. Facil..

[8] Yu H., Ma T., Wang D., Wang Z., Lv S. (2020). Review on China’s pavement engineering research 2020. China J. Highw. Transp..

[9] Guo Y. (2015). The Asphalt of Carpet of Mix Design Performance and Evaluation. M.D. Thesis.

[10] Dong Y., Liu Q., Cao D., Zhang Y. (2015). Development and application of modified asphalt dedicated to Rollpave. J. Highw. Transp. Res. Dev..

[11] Feng Z. (2016). Mechanical Behavior of Separable Precast Airfield Pavement Applied to the Desert Highway. M.D. Thesis.

[12] Dai S. (2019). Study on the Performance of Curling Prefabricated Noise Reduction Pavement. M.D. Thesis.

[13] Ye Z., Xiong H., Wang L. (2019). Collecting comprehensive traffic information using pavement vibration monitoring data. Comput. Civ. Infrastruct. Eng..

[14] Hou Y., Li Q., Zhang C., Lu G., Ye Z., Chen Y., Wang L., Cao D. (2021). The State-of-the-Art Review on Applications of Intrusive Sensing, Image Processing Techniques, and Machine Learning Methods in Pavement Monitoring and Analysis. Engineering.

[15] Fedele R., Praticò F.G., Pellicano G. (2019). The prediction of road cracks through acoustic signature: Extended finite element modeling and experiments. J. Test. Eval..

[16] Barriera M., Pouget S., Lebental B., Van Rompu J. (2020). In Situ Pavement Monitoring: A Review. Infrastructures.

[17] Dan H.C., Yang D., Zhao L.H., Wang S.P., Zhang Z. (2020). Meso-scale study on compaction characteristics of asphalt mixtures in Superpave gyratory compaction using SmartRock sensors. Constr. Build. Mater..

[18] Dong Y. (2015). Study on Material Properties and Construction Technologies of Rollable Prefabricated Asphalt Pavement. Ph.D. Thesis.

[19] Huang X., Zheng J., Feng D. (2019). Road Subgrade and Pavement Engineering.

[20] Liao G., Huang X. (2019). The Application of Abaqus Finite Element Software in Road Engineering.

[21] Liu P., Xing Q., Wang D., Oeser M. (2017). Application of Dynamic Analysis in Semi-Analytical Finite Element Method. Materials.

